# Coherent diffraction of single Rice Dwarf virus particles using hard X-rays at the Linac Coherent Light Source

**DOI:** 10.1038/sdata.2016.64

**Published:** 2016-08-01

**Authors:** Anna Munke, Jakob Andreasson, Andrew Aquila, Salah Awel, Kartik Ayyer, Anton Barty, Richard J. Bean, Peter Berntsen, Johan Bielecki, Sébastien Boutet, Maximilian Bucher, Henry N. Chapman, Benedikt J. Daurer, Hasan DeMirci, Veit Elser, Petra Fromme, Janos Hajdu, Max F. Hantke, Akifumi Higashiura, Brenda G. Hogue, Ahmad Hosseinizadeh, Yoonhee Kim, Richard A. Kirian, Hemanth K.N. Reddy, Ti-Yen Lan, Daniel S.D. Larsson, Haiguang Liu, N. Duane Loh, Filipe R.N.C. Maia, Adrian P. Mancuso, Kerstin Mühlig, Atsushi Nakagawa, Daewoong Nam, Garrett Nelson, Carl Nettelblad, Kenta Okamoto, Abbas Ourmazd, Max Rose, Gijs van der Schot, Peter Schwander, M. Marvin Seibert, Jonas A. Sellberg, Raymond G. Sierra, Changyong Song, Martin Svenda, Nicusor Timneanu, Ivan A. Vartanyants, Daniel Westphal, Max O. Wiedorn, Garth J. Williams, Paulraj Lourdu Xavier, Chun Hong Yoon, James Zook

**Affiliations:** 1 Laboratory of Molecular Biophysics, Department of Cell and Molecular Biology, Uppsala University, Husargatan 3 (Box 596), Uppsala SE-75124, Sweden; 2 Institute of Physics ASCR, v.v.i. (FZU), ELI-Beamlines Project, Prague 182 21, Czech Republic; 3 SLAC National Accelerator Laboratory, 2575 Sand Hill Road, Menlo Park, California 94025, USA; 4 Center for Free Electron Laser Science, Deutsches Elektronen-Synchrotron DESY, Hamburg 22607, Germany; 5 European XFEL GmbH, Holzkoppel 4, Schenefeld 22869, Germany; 6 Australian Research Council Centre of Excellence in Advanced Molecular Imaging, La Trobe Institute for Molecular Science, La Trobe University, Melbourne 3086, Australia; 7 Argonne National Laboratory, 9700 South Cass Avenue, Argonne, Illinois 60439, USA; 8 Institut für Optik und Atomare Physik, Technische Universität Berlin, Hardenbergstraße 36, Berlin 10623, Germany; 9 Department of Physics, University of Hamburg, Hamburg 22761, Germany; 10 Stanford PULSE Institute, 2575 Sand Hill Road, Menlo Park, California 94025, USA; 11 Laboratory of Atomic and Solid State Physics, Cornell University, Ithaca, New York 14853, USA; 12 Arizona State University, School of Molecular Sciences (SMS), Tempe, Arizona 85287-1604, USA; 13 Biodesign Center for Applied Structural Discovery, Biodesign Institute at Arizona State University, Tempe 85287, USA; 14 Institute for Protein Research, Osaka University, Suita, Osaka 565-0871, Japan; 15 Arizona State University, School of Life Sciences (SOLS), Tempe, Arizona 85287-5401, USA; 16 Biodesign Center for Infectious Diseases and Vaccinology, Biodesign Institute at Arizona State University, Tempe 85287, USA; 17 Department of Physics, University of Wisconsin Milwaukee, 3135 North Maryland Ave., Milwaukee, Wisconsin 53211, USA; 18 School of Materials Science and Engineering, Gwangju Institute of Science and Technology, Gwangju 61005, Korea; 19 Arizona State University, Department of Physics, Tempe, Arizona 85287, USA; 20 Beijing Computational Science Research Center, 8 W Dongbeiwang Rd, Haidian, Beijing 100193, China; 21 Centre for Bio-imaging Sciences, National University of Singapore, 14 Science Drive 4, BLK S1A, Singapore 117543, Singapore; 22 Department of Physics, Pohang University of Science and Technology, Pohang 37673, Korea; 23 Department of Information Technology, Science for Life Laboratory, Uppsala University, Lägerhyddsvägen 2 (Box 337), Uppsala SE-75105, Sweden; 24 Deutsches Elektronen-Synchrotron DESY, Notkestraße 85, Hamburg D-22607, Germany; 25 Biomedical and X-Ray Physics, Department of Applied Physics, AlbaNova University Center, KTH Royal Institute of Technology, Stockholm SE-106 91, Sweden; 26 Department of Physics and Astronomy, Uppsala University, Lägerhyddsvägen 1 (Box 516), Uppsala SE-75120, Sweden; 27 National Research Nuclear University MEPhI (Moscow Engineering Physics Institute), Kashirskoe shosse 31, Moscow 115409, Russia; 28 Brookhaven National Laboratory, NSLS-II, Upton, New York 11973, USA; 29 Max-Planck Institute for the Structure and Dynamics of Matter, CFEL, Hamburg 22607, Germany

**Keywords:** Structural biology, Imaging, Single-molecule biophysics, Biological physics

## Abstract

Single particle diffractive imaging data from Rice Dwarf Virus (RDV) were recorded using the Coherent X-ray Imaging (CXI) instrument at the Linac Coherent Light Source (LCLS). RDV was chosen as it is a well-characterized model system, useful for proof-of-principle experiments, system optimization and algorithm development. RDV, an icosahedral virus of about 70 nm in diameter, was aerosolized and injected into the approximately 0.1 μm diameter focused hard X-ray beam at the CXI instrument of LCLS. Diffraction patterns from RDV with signal to 5.9 Ångström were recorded. The diffraction data are available through the Coherent X-ray Imaging Data Bank (CXIDB) as a resource for algorithm development, the contents of which are described here.

## Background & Summary

For several decades, X-ray crystallography has been the dominant technique to solve the three-dimensional (3D) structure of biological macromolecules at atomic resolution. Structures of proteins, protein complexes and the machinery of entire biological reaction pathways have been elucidated, leading to numerous breakthroughs in our understanding of molecular architecture and function. However, not every protein complex crystallizes, a necessary condition for investigation using these methods. Radiation damage additionally limits the resolution for biological objects, both crystalline and non-crystalline, which for high exposures leads to the determination of structures representative of a photodamaged state^1–3^. The ultrashort and extremely bright pulses from X-ray free electron lasers (XFELs) were predicted to outrun radiation damage processes and allow the recording of diffraction data from samples prior to any significant motion of the nuclei occurring^2^. This has been experimentally demonstrated at nanometer resolution in isolated objects^4–8^ and Ångström (Å) resolution in micro/nanocrystals^9,10^. Yet the goal of near-atomic resolution single particle imaging, using X-rays, remains elusive.

The single particle imaging (SPI) initiative is a large collaborative team of researchers from several institutions formed to identify and solve the challenges required for high resolution imaging with XFELs. The aim of the SPI initiative, as laid out in a published roadmap^11^, is to establish a community-wide approach to take up the scientific and technical challenges of single-molecule imaging with X-rays. In addition to developing solutions to the technical challenges, a critical part of this project was selecting a well-characterized model system needed for demonstration experiments. After considering homogeneity, uniform size distribution, particle concentration, having a known structure, and the ability to be aerosolized for injection into the XFEL beam, Rice Dwarf Virus (RDV) was selected for the first experiment (see methods) in this initiative.

RDV is an icosahedral RNA virus of about 70 nm in diameter, and is the causative agent of rice dwarf disease. This disease creates severe economic damage in China, Japan and other Asian countries due to speck formation and its destructive effects on plant growth for rice, wheat, barley, and other gramineae plants. Leafhopper insects are the primary host in which the virus particles replicate and from which they are then transmitted to leaves. The virus particles consist of two shells, an inner and an outer capsid, enclosing a double stranded RNA genome. The genome encodes 12 products, seven of which are considered structural proteins. A thin layer of P3 capsid proteins^12^ makes up the inner capsid and three proteins of mainly P8 (ref. 13), but also P2 (ref. 14) and P9 (ref. 15), form the outer capsid. Found in the core, together with the genome, are P1 (putative RNA polymerase^16^), P5 (putative guanylyltransferase^17^) and P7 (a nonspecific nucleic acid binding protein^18^). A 3D structure of the capsid was previously solved by X-ray crystallography at 3.5 Å resolution (PDB 1UF2)^19^.

**Figure 1:**
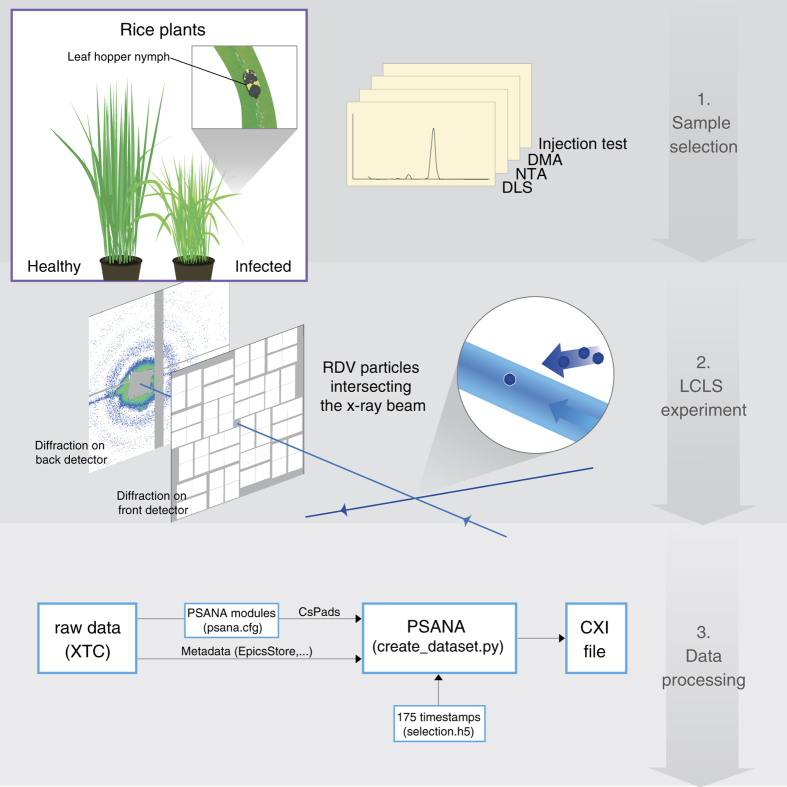
Experimental design. (1) As the first step in the experiment, an analysis of candidate samples was carried out and a primary target (Rice Dwarf Virus, RDV) was selected. RDV was purified from grasshopper nymphs, which were fed infected rice plants as described in the text. (2) Purified virus particles were then injected into the X-ray beam of the LCLS and diffraction patterns were recorded on the front and back detectors of the CXI instrument^20^. (3) The raw data were pre-processed using *psana*^30^ and converted to XTC files. 175 frames of strong hits were selected and converted into the CXI file form.

RDV was aerosolized and delivered into the hard X-ray Coherent X-ray Imaging (CXI) nanofocus instrument at Linac Coherent Light Source (LCLS)^20,21^ using an aerodynamic lens injector^22^. Diffraction patterns were recorded at a rate of 120 Hz using two Cornell-SLAC Pixel Array Detectors (CSPAD), a large 2.3 Mpix detector located close to the sample for wide angle scattering and another smaller 0.14 Mpix ‘2×2’ detector located further downstream to detect small angle scattering^23,24^ (pictorially shown in center panel of Fig. 1). The photon energy was 7 keV, the pulse duration was <50 fs, and the average pulse energy immediately downstream of the undulator was 4 mJ. (see Methods for a detailed description).

In the data deposited in the Coherent X-ray Imaging Data Bank (CXIDB)^25^, we record diffraction from RDV particles hit by the LCLS X-ray pulse on the back detector. Also recorded is elevated scattering on the front detector for ‘hits', that are identified based on the data on the back detector. This indicates that measurable photons are recorded from the sample up to a scattering angle commensurate with 5.9 Å resolution.

## Methods

### Sample preparation

Nymphs of *Nephotettix nigropictus* were fed on diseased rice plants. The purification procedure of the RDV O strain^26^ followed the procedure of Omura *et al.*^27^ with modified sucrose concentrations as follows. A meat chopper was used to grind the infected rice leaves and the resultant slurry was treated with CCl_4_ and subjected to repeated precipitations and consecutive density gradient centrifugations in 40 to 60% and 40 to 70% sucrose. The pellet from the final centrifugation of the viral particle band was resuspended in a 0.1 M solution of histidine that contained 0.01 M MgCl_2_ (pH 6.2). The sample contained all viral components except the P2 protein, which was removed by the CCl_4_ treatment. This removal prevents infection through oral intake by the insect and direct injection is instead required for vector infection.

### Pre-characterization experiments

**Figure 2:**
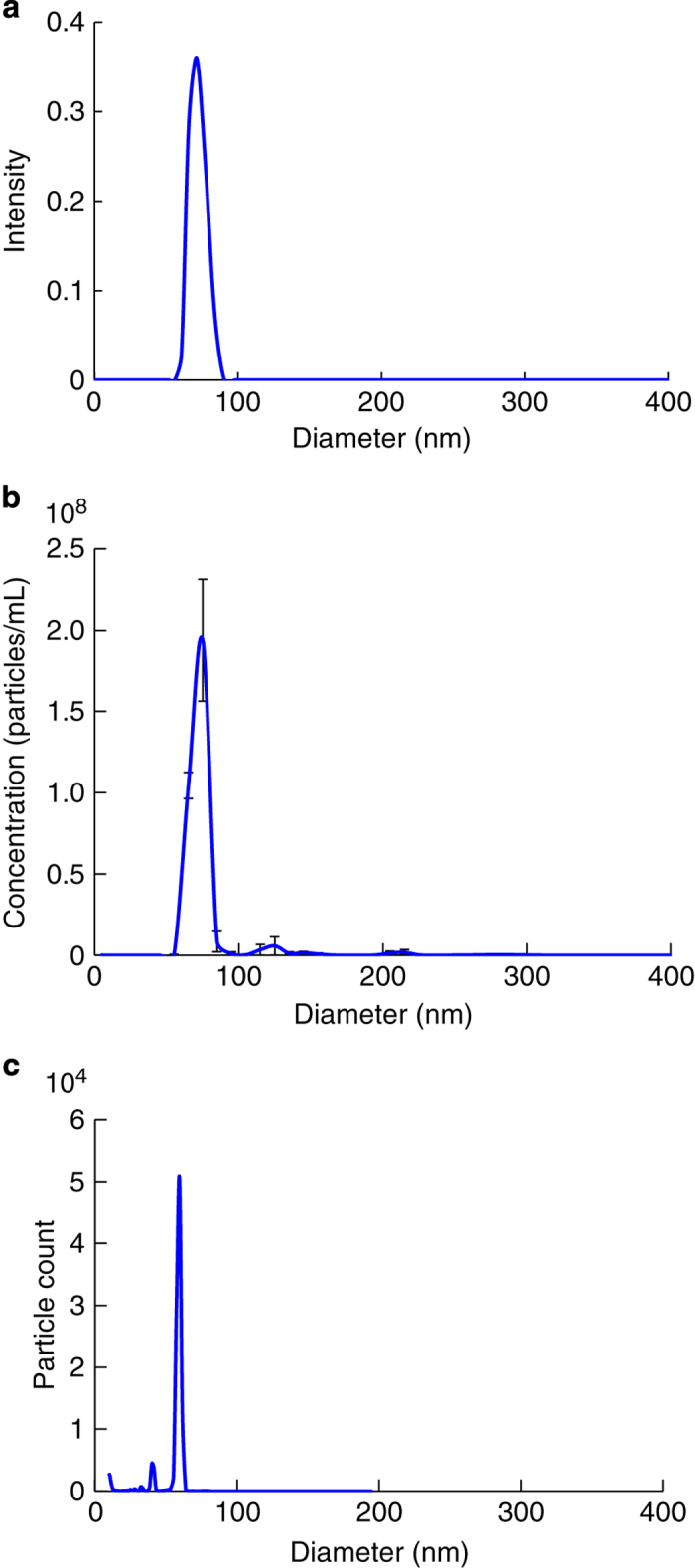
The purity of RDV was analyzed with DLS, NTA and DMA. (**a**), Size distribution determined with DLS. The average size of the RDV particles was approximately 76 nm. (**b**), Size distribution determined with NTA. The mean and mode of the RDV particles were approximately 76 and 73 nm, respectively. (**c**), DMA measurement of RDV gave an apparent particle diameter of approximately 60 nm. Here the actual gas flow was lower than the set value, which resulted in an under estimation of the particle size by about 10%. The main purpose of the DMA measurement was to assess sample purity and the results show that.

Reference samples need to have a known structure, be available at high concentration, exhibit monodisperse size distribution and be compatible with available sample delivery techniques. Pre-characterization was performed using Dynamic Light Scattering (DLS), Nanoparticle Tracking Analysis (NTA), Differential Mobility Analysis (DMA) and aerosol injection testing as shown in Fig. 2 (Data Citation 1). RDV was determined to satisfy these requirements and was selected as the test sample for the LCLS experiment.

#### Injection testing

The sample was injected using an injection setup identical to that used in the LCLS experiment (see *Sample injection at the LCLS*) to investigate their ability to aerosolize and their resistance to the injection procedure. By placing a microscope glass slide covered by a gel piece (Gel-Pak) beneath the outlet of the aerodynamic lens (at the same position as the interaction region with the X-ray beam in the subsequent LCLS experiments), a particle dust could be observed through an objective lens mounted below. In a second set of experiments a formvar/carbon grid (#01754-F, F/C 400 mesh Cu, Ted Pella Inc.) was substituted for the glass slide, to capture RDV particles that had traversed the injector. These samples were examined further using an environmental scanning electron microscope (ESEM) (Quanta FEG 650, FEI). The pressure in the vacuum chamber was kept at approximately 10^−5^ mBar.

#### Sample size and monodispersity in the liquid phase

The size distribution of the RDV sample in solution (250 mM ammonium acetate buffer, pH 7.5) was measured using both DLS (w130i, AvidNano Ltd. and Spectrolight 600, Molecular Dimensions) and NTA techniques (NanoSight, model LM10, Malvern Instruments Ltd.). For these DLS and NTA measurements, the sample was diluted to 10^9^ particles ml^−1^ and 10^8^ particles ml^−1^, respectively. The measured size distribution is shown in Fig. 2a,b.

#### Sample size and monodispersity in the gas phase

The size distribution of the RDV sample in the gas phase were measured by means of Electrophoretic DMA. RDV was aerosolized with a nano-Electrospray ionization (ESI) source (TSI model 3480) and passed through an electrostatic classifier (TSI model 3480) whose size selection window was continuously scanned. Transmitted particles were counted with a condensation particle counter (CPC, TSI model 3786). The size distribution is shown in Fig. 2c.

### Sample injection at LCLS

The experiment was carried out at the CXI instrument at the LCLS^19,20^. An aerosol injector (described in ref. 8) was used to introduce the particles into the X-ray beam. Purified RDV were transferred to a volatile buffer (250 mM ammonium acetate, pH 7.5) at a concentration of 10^12^ particles ml^−1^ and introduced to the injector via a gas dynamic virtual nozzle (GDVN)^28^ at a flow rate of 1–2 μl min^−1^. The aerosol passed through a skimmer and a relaxation chamber and it was focused into a narrow particle beam by an aerodynamic lens. By regulating gas, liquid flow and skimmer pressure, the quality of the particle beam could be optimized. Injected particles intersected the X-ray beam in random orientations.

### Experimental setup and data collection

Data were collected at the CXI instrument at LCLS. LCLS was tuned to a photon energy of 7 keV and produced pulses with approximately 4 mJ pulse energy and <50 fs duration. The selection of the photon energy, within the CXI operation range of 5 to 11 keV, was driven by the competition between the sample’s scattering cross section, which tends to be high at lower energies, and the ability to discriminate single-photon events in the detector, which increases at the high end of the range. Additionally, photon energies below the Iron K-alpha excitation edge at 7.1 keV avoid an isotropic fluorescence signal from the steel walls of the vacuum chamber, which in turn would complicate the identification of photons scattered by the sample.

X-rays were focused using a pair of Kirkpatrick-Baez (KB) mirrors to a nominal size of 0.1×0.1 μm. The focused beam passed through a set of beam-defining apertures to reduce the X-ray scattering imperfections in the optical system as the FEL beam overfills the KB entrance. Additional cleanup slits and apertures, including a post sample aperture, are used to limit background scatter. The post sample aperture limited the collection angle to commensurate with 5.9 Å resolution. The post sample aperture is a small 3 mm circular aperture positioned just downstream of the interaction region and is used to prevent scattered X-rays coming from locations other than the sample position reaching the sensitive surface of the detector. Small angle diffraction patterns were recorded with a CSPAD 0.14 Mpix detector (also referred to as the back detector), located 2.4 m downstream of the interaction and high-angle scattering was captured on a 2.3 Mpix CSPAD detector (also referred to as the front detector), located 217.4 mm downstream from the interaction point, in a tandem arrangement as shown in the center panel of Fig. 1 (refs 23,^24^). All data events were recorded and synchronized with the LCLS repetition rate of 120 Hz. The back detector was offset with respect to the optical axis of the focusing optics, and extended to a maximal resolution of c. 15.2 nm and c. 11.6 nm on the edge and in the corner, respectively. A semitransparent beam-stop, consisting of 25 μm Ti and 100 μm Zn, was utilized so that very low-q scattering could be collected, as well as provide a monitor for the direct beam. Data were analyzed onsite using *Hummingbird*, a fast online analysis tool developed for single particle imaging^29^ and *Cheetah*, a software for high-throughput reduction and analysis of serial femtosecond X-ray diffraction data^30^.

### Data processing steps used

To provide interpretable data in addition to the raw XTC files, the native format of the LCLS data stream, we selected a small subset of diffraction events (175 frames) and converted them into a CXI file using *psana*^31^. For both back and front detectors, data were calibrated using psana’s *ImgAlgos.NDArrCalib* module, with pedestal subtraction (*do_peds*), common-mode correction (do_cmod), statistical correction (do_stat) and gain corrections (do_gain) turned on. Pixel gains were calculated by generating per-pixel histograms from a flat-field run and fitting a bimodal distribution with respect to the noise peak and the single photon peak. Using psana’s *CSPadPixCoords.CSPadImageProducer* and *CSPadPixCoords.CSPad2x2ImageProducer*, the detector panels were assembled in order to form real images. For a list of provided files and data entries, see section Data Records. The 175 patterns were selected manually by means of identifying strong diffraction signal showing similarities to simulations. See Technical Validation for more details.

## Data Records

### Data citation 1—Sample size and monodispersity

The data are available at Figshare (Data Citation 1) and contains an excel file with raw data from the DLS, NTA and DMA measurements.

### Data citation 2—Coherent diffractive imaging data

The data are deposited in the CXIDB^25^ (Data Citation 2) and stored in the CXIDB data format, which is based on the HDF5 format. HDF5 files are readable in many computing environments, including Python using the h5py module and MATLAB using e.g., the h5read function. Convenient functions for accessing the CXIDB data file exist in the libspimage package for C and Python^32^. For visualizing data in the CXIDB format, the *Owl* software is convenient (https://github.com/FilipeMaia/owl/). In addition to the CXI file, the conversion script (create_dataset.py) and additional metadata files (selection.h5, psana.cfg) are provided along with usage instructions. Detector panel calibration files mapping data to real space are also provided. Configuration files for *Hummingbird*, *psana* and *Cheetah* are provided for completeness of describing processing performed on the deposited data. Table 1 describes the files deposited in the CXIDB.

## Technical Validation

### Background scattering and direct beam scatter

**Figure 3:**
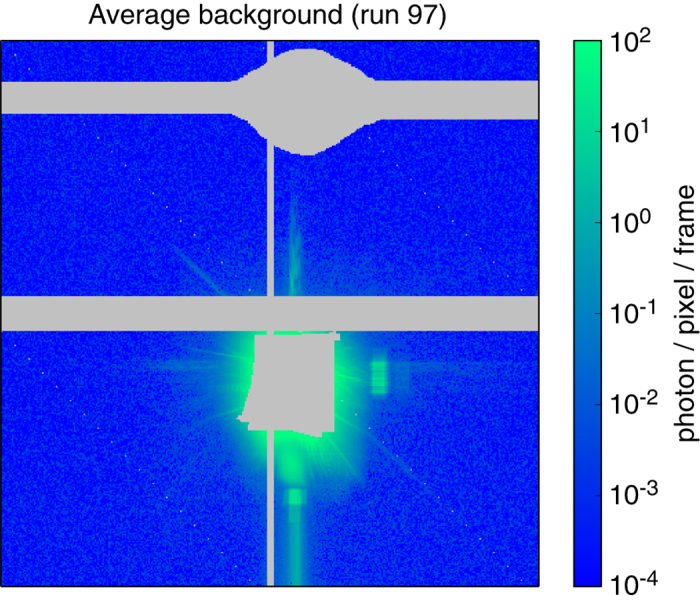
Background image of the back detector, averaged from 1000 non-hits and non-dark frames. The grey areas are masked out. They correspond to the beamstop, the gap between sensors, and a shadow mask of an additional beamstop from the beamstop holder. Unbonded pixels that do not read out signal, are likewise masked out.

A background scattering pattern was derived by averaging 1000 frames that did not include any hits or dark frames. This background, as well as suggested masks for non-responsive pixels and beamstops, are shown in Fig. 3.

**Figure 4:**
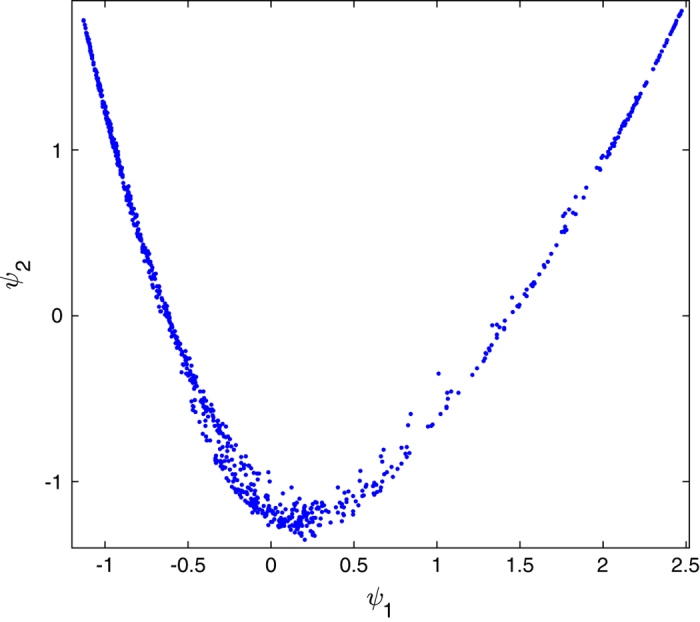
Manifold of raw back detector data in two dimensions. Each point represents a diffraction pattern. The axes are orthogonal coordinates provided by the manifold embedding algorithm^37^.

Additionally, for the back detector data, manifold-embedding methods were used to detect and identify the nature and origin of stochastic changes, and quantify the necessary corrections to the background. The manifold of raw RDV single-particle snapshots is shown in Fig. 4, where each point represents a diffraction pattern^33–35^. The parabolic nature of this manifold reveals that a single parameter dominates the changes from snapshot to snapshot, namely fluctuations in pulse intensity, consistent with the self-amplified spontaneous emission process of the FEL. This can be corrected by appropriate normalization procedures^36^. In addition to a monotonic intensity change along the parabola, a prominent deviation is evident. This is caused by a shift of about one pixel, of the center of intensity along the lateral direction of the beam. The cause of this shift is a drift in pointing of the offset mirrors on the beam-defining aperture of the KB mirrors for the CXI beamline.

### Simulated diffraction data of expected size

**Figure 5:**
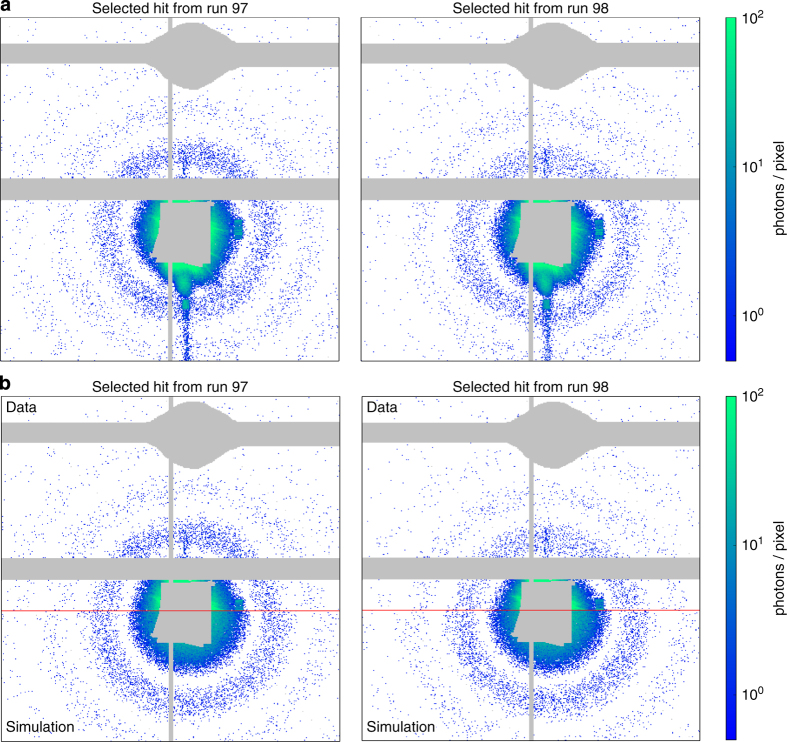
Measured and simulated diffraction patterns of single RDV particles. (**a**), Two hits on the back detector, selected on the expected size for RDV and high diffraction intensity. (**b**), Measured and simulated data combined. The top half of each of the two patterns shows the measured signal and is identical to the top halves of the patterns in (**a**). The bottom half of each pattern shows simulated diffraction data from a homogenous sphere of size 71 nm and with a mass density of 1.381 gcm^−3^. The simulation assumes a photon energy of 7 keV, a detector distance of 2.4 m, a pixel size of 110 microns and a conversion of 33 ADUs per photon. Regions of beam-stops and gaps between the detector panels are masked in grey.

In Fig. 5, two diffraction patterns from different single particle hits are shown in comparison to simulated diffraction from homogeneous spheres of size 71 nm. In the simulation, a photon energy of 7 keV and an assumed mass density for RDV of 1.381 gcm^−3^ was used. The back detector was simulated using a detector distance of 2.4 m, a pixel size of 110 microns and a signal conversion rate of 33 ADUs per photon.

### Signal above background on front detector

**Figure 6:**
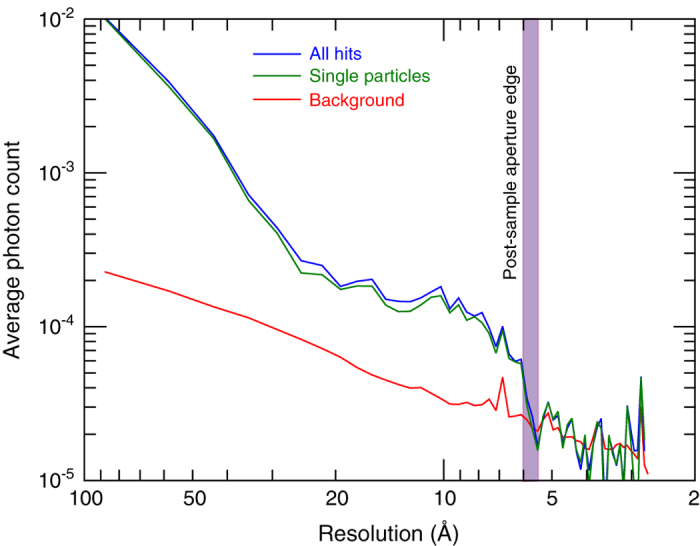
Radial average of signal on the front detector from blank frames compared to radial average from frames determined to be hits. Elevated photon counts from the sample are visible up to an angle commensurate with 5.9 Å resolution, this being the resolution limit set by the angular acceptance of the post-sample aperture. ‘All hits’ is the average of all hits and includes all particles independent of size (including clusters of particles), while ‘single particle’ is the average of hits that are of the appropriate size to be isolated single particles.

The front detector was located 217.4 mm downstream of the sample interaction region and collected diffraction data extending to a resolution of 5.9 Å. Although hits are immediately apparent on the back detector, and this signal is used for hit finding, determining whether there is useful signal from the sample on the front detector above background levels is not immediately apparent from any individual image. A radial average of the sum of frames determined by *Cheetah* to be hits shows that there is indeed consistently elevated signal above background when sample is detected in the beam based on the back detector (Fig. 6). This intensity distribution falls off with the expected q-dependence, and stops at a resolution of 5.9 Å. This resolution limit is set by the angular acceptance of the post-sample aperture. Beyond this resolution both radial sums are identical, further supporting the notion that signal up to 5.9 Å resolution comes from individual particles. This validates the potential usefulness of signal on the front detector for image analysis. *Cheetah* processing scripts are included in the archive.

### Validation that scattering comes from RDV

The method of least surprise (described below) was used to determine whether signal on the front detector corresponded to the expected signal from RDV.

Data were converted from Analog-to-Digital Units (ADU) measured by the detector into photon counts using the relation

$$k_{i}=ceil[(A_{i}-0.5\gamma )/\gamma ]$$

where *k*_*i*_ is the photon count at pixel *i*, *A*_*i*_ is the dark, common-mode, gain-corrected ADU measured at pixel *i*, and *γ* is the average ADUs per photon for the detector which was calculated from a flat-field run. In this analysis, we use front detector data up to 6.67 Å resolution, which corresponds to a radius of 265 pixels.

Assuming Poisson statistics, we define the surprise function as the negative log-likelihood

$$S(K;\Phi ,\Omega_{j})=-\sum_{i=1}^{N}\text{log}(\frac{n_{i}^{k_{i}}e^{-n_{i}}}{k_{i}!})\equiv -\sum_{i=1}^{N}\text{log}\;P(n_{i},k_{i})$$

where *K* denotes the dependence on data, with *k*_*i*_ being the measured photon count at pixel *i*, *n*_*i*_ is the average photon number at pixel *i* when the fluence is Φ and the RDV particle has orientation Ω_*j*_, and the summation runs over all the pixels. Minimizing the surprise function, or maximizing the log-likelihood, across different orientations and fluence values, we assign each data frame with the orientation and fluence at which it was most likely recorded. To help us assess the quality of these assignments, we further ‘normalize’ the surprise function. Given the RDV model (PDB 1UF2)^19^ with an estimate of the particle orientation and fluence, we calculate the mean

$$〈S(\Phi ,\Omega_{j})〉=-\sum_{i=1}^{N}〈\text{log}\;P(n_{i},k)〉$$

and the standard deviation

$$\sigma_{S}(\Phi ,\Omega_{j})={\left[\sum_{i=1}^{N}〈{\left(\text{log}\;P(n_{i},k)\right)}^{2}〉-{〈\text{log}\;P(n_{i},k)〉}^{2}\right]}^{1/2}$$

of the surprise function, where 〈⋯〉 denotes the expectation value under the Poisson distribution. Note that $〈S(\Phi ,\Omega_{j})〉$ and *σ*_*S*_(Φ, Ω_*j*_) are independent of the data *K*. The normalized surprise function, or its z-score,

$$z(K;\Phi ,\Omega_{j})\equiv \frac{S(K;\Phi ,\Omega_{j})-〈S(\Phi ,\Omega_{j})〉}{\sigma_{S}(\Phi ,\Omega_{j})}$$

measures the agreement of the data with a known model: The data are inconsistent with the model when the absolute value of the z-score is much greater than unity: a z-score much greater than unity is consistent with the data being ‘surprising’ given the assumed model.

**Figure 7:**
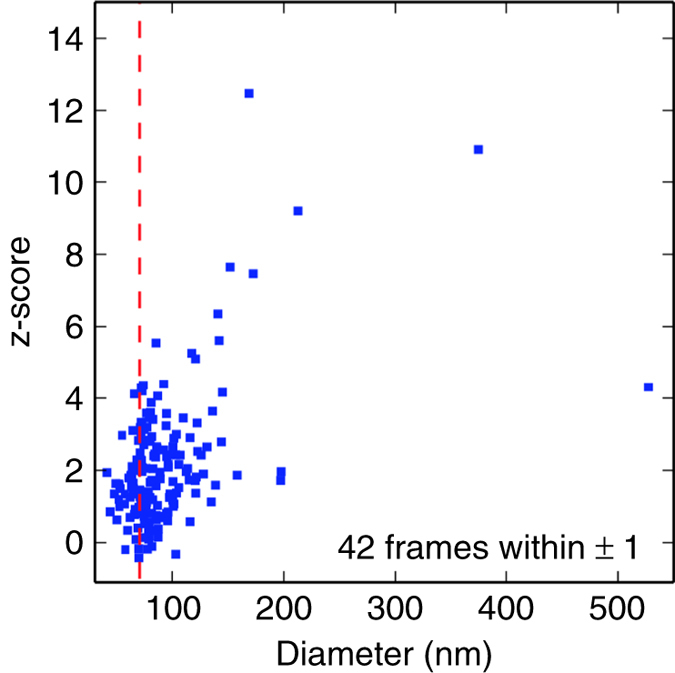
Front detector normalized surprise (z-score) versus back detector particle size fits. The dashed red line indicates the diameter (70.8 nm) of the RDV model. The normalized surprise function, or its z-score, measures the agreement of the data with a known model: The data are inconsistent with the model when the absolute value of the z-score is much greater than unity: a z-score much greater than unity is consistent with the data being ‘surprising’ given the assumed model.

The z-scores of all the selected frames versus particle size are shown in Fig. 7. The particle sizes were determined by fitting back detector data to a homogeneous sphere model with adjustable size. Frames with particle size close to the diameter (70.8 nm) of the RDV model generally have smaller z-scores, though some still manifest inconsistency with the model. The source of this could be the presence of a water layer on the particle surface. This model-based surprise function calculation may potentially be useful for hit-finding, especially when the signal is as weak as the front detector data.

## Usage Notes

The dataset (CXIDB ID 36) contains the full data stream recorded during the experiment in.xtc format. The dataset also contains a set of pre-selected hits as a CXI file (as described above) from both CSPAD detectors plus instrument metadata. XTC files are the native format of LCLS and can be read using analysis frameworks provided by the LCLS (see https://confluence.slac.stanford.edu/display/PSDM/LCLS+Data+Analysis).

## Additional Information

**How to cite this article:** Munke, A. *et al.* Coherent diffraction of single Rice Dwarf virus particles using hard X-rays at the Linac Coherent Light Source. *Sci. Data* 3:160064 doi: 10.1038/sdata.2016.64 (2016).

## Supplementary Material



## Figures and Tables

**Table 1:** Deposited data and configuration files.

Experimental data		
Data type	Example filename	File format
Raw data (all exposures)	e625-r0002-s00-c00.xtc	XTC
Diffraction Pattern (only selected hits)	cxidb-36.cxi	CXI
		
Metadata		
Data type	Filename	File format
Cheetah initialization file (preprocessing)	cheetah.ini	Text
Cheetah configuration file (preprocessing)	psana.cfg	Text
Bad pixel mask (*front* detector)	mask_cspad.h5	Hdf5
Bad pixel mask (*back* detector)	mask_cspad2x2.h5	Hdf5
Conversion script	create_dataset.py	Python
File containing the timestamps of the selected hits	selection.h5	Hdf5
		
Cheetah preprocessing		
Data type	Folder name	File format
Calibration data	calib	various
Cheetah GUI related files	gui	various
Hdf5 files of dark runs and hits	hdf5	Hdf5
Indexing files	indexing	various
Cheetah preprocessing related scripts	process	various
This table describes the files deposited in the CXIDB under accession number ID 36. ID 36 consists of Experimental data, *Cheetah* preprocessing files, and metadata.		
